# RNA binding protein 24 regulates the translation and replication of hepatitis C virus

**DOI:** 10.1007/s13238-018-0507-x

**Published:** 2018-01-30

**Authors:** Huang Cao, Kaitao Zhao, Yongxuan Yao, Jing Guo, Xiaoxiao Gao, Qi Yang, Min Guo, Wandi Zhu, Yun Wang, Chunchen Wu, Jizheng Chen, Yuan Zhou, Xue Hu, Mengji Lu, Xinwen Chen, Rongjuan Pei

**Affiliations:** 1 State Key Laboratory of Virology, Wuhan Institute of Virology, Chinese Academy of Sciences, Wuhan, 430071 China; 2 University of Chinese Academy of Sciences, Beijing, 100049 China; 3 Department of Infectious Disease, University Hospital Essen, University of Duisburg-Essen, Gebäude V15, 45117 Essen, Germany

**Keywords:** RNA binding protein, RBM24, hepatitis C virus, translation, replication

## Abstract

The secondary structures of hepatitis C virus (HCV) RNA and the cellular proteins that bind to them are important for modulating both translation and RNA replication. However, the sets of RNA-binding proteins involved in the regulation of HCV translation, replication and encapsidation remain unknown. Here, we identified RNA binding motif protein 24 (RBM24) as a host factor participated in HCV translation and replication. Knockdown of RBM24 reduced HCV propagation in Huh7.5.1 cells. An enhanced translation and delayed RNA synthesis during the early phase of infection was observed in RBM24 silencing cells. However, both overexpression of RBM24 and recombinant human RBM24 protein suppressed HCV IRES-mediated translation. Further analysis revealed that the assembly of the 80S ribosome on the HCV IRES was interrupted by RBM24 protein through binding to the 5′-UTR. RBM24 could also interact with HCV Core and enhance the interaction of Core and 5′-UTR, which suppresses the expression of HCV. Moreover, RBM24 enhanced the interaction between the 5′- and 3′-UTRs in the HCV genome, which probably explained its requirement in HCV genome replication. Therefore, RBM24 is a novel host factor involved in HCV replication and may function at the switch from translation to replication.

## Introduction

The genome of hepatitis C virus (HCV) is composed of a single open reading frame (ORF) flanked by 5′- and 3′-untranslated regions (UTRs). The ORF encodes a polyprotein of approximately 3,000 amino acids (aa) that is processed into viral structural and non-structural proteins by host and viral proteinases. Like other positive-strand RNA viruses, the genomic RNA of HCV is believed to serve as the template for translation and negative-strand RNA synthesis (Ranjith-Kumar and Kao, 2006). The same RNA cannot serve as template for both processes simultaneously because translation proceeds in the 5′ to 3′ direction, whereas negative-strand RNA synthesis occurs in the 3′ to 5′ direction (Shi and Lai, 2006). Thus the control of translation and replication during the initial period post-infection is essential for viral proliferation.

The 5′- and 3′-UTRs are the most conserved regions of HCV RNA among the different genotypes and isolates. Both of these regions form complex secondary structures with multiple stem-loops and are involved in the control of translation and RNA replication (Shi and Lai, 2006). The 5′-UTR where the internal ribosome entry site (IRES) located within is the key element that mediates HCV translation initiation (Tsukiyama-Kohara et al., 1992). During ribosome complex recruitment, the core domain of IRES (domains II to IV of the 5′-UTR) directly binds the 40S subunit. Domain III interacts with 18S rRNA and subunits of eIF3 (Malygin et al., 2013; Sun et al., 2013), while domain II facilitates recruitment of the 60S ribosome and promotes translation elongation (Filbin et al., 2013; Locker et al., 2007). Besides functioning in translation, domains I and II of the 5′-UTR, corresponding with the most 3′-terminal region of the negative strand, are necessary for HCV replication (Kim et al., 2002; Schuster et al., 2002; Mahias et al., 2010; Reigadas et al., 2001; Astier-Gin et al., 2005). The 3′-UTR composed of a variable region, a polyU/UC tract of variable length and a highly conserved 98-bases element designated as 3′-X, is an essential *cis*-acting element for HCV replication, while the 3′-X region is also involved in IRES dependent translation (Bai et al., 2013; Wood et al., 2001; Shetty et al., 2010). Another *cis*-acting element functioning in both replication and translation is the stem-loop 3.2 (SL3.2) located at the 3′ end of the NS5B coding region (Cheng et al., 1999). The interaction between SL3.2 and 3′-X is necessary for replication (Friebe et al., 2005), while the interaction between SL3.2 and domain III of the 5′-UTR has been reported to inhibit IRES-mediated translation (Romero-Lopez and Berzal-Herranz, 2012, 2009), which is thought to play a crucial role in the switch between the translation and replication during the HCV life cycle.

In addition to the *cis* elements, the translation and replication of HCV is under the control of *trans*-acting factors, including viral and host factors. The nonstructural proteins form replication complex and control the RNA replication, while the Core, NS3 has also been reported to modulate IRES activity *in trans* (Shimoike et al., 1999; Ray and Das, 2011; Li et al., 2003; Shimoike et al., 2006; Tanaka et al., 2000). Among the host factors controlling HCV replication or translation *in trans*, several candidates are involved in mediating both of these processes, including human La protein, poly(rC)-binding protein 2 (PCBP2) and mice minute virus NS1-associated protein 1 (NSAP1) (Ali et al., 2000; Ito and Lai, 1997; Chang and Luo, 2006; Spangberg and Schwartz, 1999; Park et al., 2011). La protein has been reported to enhance HCV IRES-mediated translation by binding to the 5′-UTR of the HCV genome (Ali et al., 2000). Its involvement in HCV replication may be explained by the fact that it also interacts with the 3′-UTR of HCV RNA and prevents its degradation, in addition to promoting the linkage between the 5′-UTR and 3′-UTR (Kumar et al., 2013). The interplay of La protein and NS3, a viral *trans*-element, may regulate the translation-replication switch of HCV (Ray and Das, 2011). PCBP2 interacts with the 5′- and 3′-UTRs of HCV RNA and enhances HCV translation, the circularization of HCV RNA and replication (Wang et al., 2011). NSAP1 enhances HCV IRES activity by binding to the IRES near the Core start codon (Kim et al., 2004); in addition, its interaction with the 40S ribosomal subunit facilitates 80S complex formation (Park et al., 2011). NSAP1 has been suggested to play a role in HCV replication because knockdown of its expression reduces the replication of this virus (Liu et al., 2009). A common feature of these mentioned proteins above is the containing of multiple RNA recognition motifs (RRM); thus, it is of interest to analyze whether other proteins with RRMs are also involved in HCV replication.

RNA-binding proteins (RBPs), which bind to double or single-stranded RNA, play key roles in the post-transcriptional control of RNA. Previous reports have shown that RNA binding protein 24 (RBM24), which harbors a single RRM at its N-terminus, is required for cardiovascular development and myogenesis because it regulates the stability and/or alternative splicing of the mRNAs of related genes (Jin et al., 2010; Poon et al., 2012; Xu et al., 2014; Yang et al., 2014). Gene array analysis has revealed the significant up-regulation of RBM24 during HCV infection. Considering its role in the post-transcriptional control of RNA, we analyzed the function of RBM24 in the HCV life cycle and found that it binds to both the 5′- and 3′-UTRs of HCV, inhibits HCV IRES-mediated translation by interrupting 60S ribosome recruitment and promotes HCV replication by linking the HCV 5′- and 3′-UTRs.

## Results

### RBM24 participates in HCV propagation

**Figure 1:**
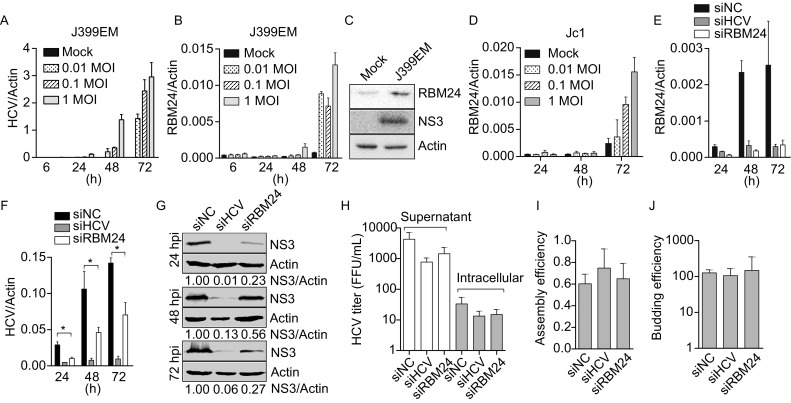
**Participation of RBM24 in HCV life cycle**. (A–C) Huh7.5.1 cells infected with J399EM of different MOIs (0, 0.01, 0.1 and 1) were harvested at the indicated time points. (A and B) The intracellular HCV RNA levels (A) and RBM24 mRNA levels (B) were quantified by qRT-PCR. The protein levels of HCV NS3 (0.1 MOI) and endogenous RBM24 were detected by WB at 72 hpi (C). (D) The RBM24 mRNA levels in Jc1 infected Huh7.5.1 cells were quantified by qRT-PCR. (E–H) Huh7.5.1 cells were transfected with the indicated siRNAs and then infected with Jc1 at 1 MOI. The intracellular RBM24 mRNA (E) levels and HCV RNA (F) levels were quantified by qRT-PCR at the indicated time point. (G) The NS3 protein expression levels were detected by WB. (H) The viral titers in supernatant and in cells at 72 hpi were monitered. (I and J) The assembly efficiency (the ratio of the number of supernatant HCV RNA copies to that of intracellular HCV RNA copies) and budding efficiency (the ratio of the HCV titer in the cell supernatant to that in cells) were calculated

Several host factors with RNA-binding domains have been previously reported to be involved in the control of HCV translation and/or replication. RBM24, which has been shown to be up-regulated by HCV infection by microarray analysis, was selected for further study. The up-regulation of RBM24 by HCV infection was first validated at the RNA and protein levels. As shown in Fig. 1A–D, the RBM24 mRNA level was significantly up-regulated in time- and viral dose-dependent manners by J399EM and Jc1 infection, and its protein expression level was also increased by HCV infection (Fig. 1C).

The involvement of RBM24 in the HCV life cycle was then analyzed. Huh7.5.1 cells were transfected with siRNAs, including nonspecific siNC, siRBM24 targeting exon 2 of RBM24 and siHCV targeting the HCV IRES, and then were infected with Jc1. siHCV successfully restricted HCV replication, and it also inhibited the up-regulation of RBM24, further confirming that RBM24 expression was elevated by HCV infection (Fig. 1E and 1F). siRBM24 efficiently decreased the mRNA level of RBM24 (Fig. 1E) without significantly influencing cell viability. The silencing of RBM24 considerably reduced the HCV RNA level by approximately 70% at 24 hpi, and by 50% at 48 hpi and 72 hpi, respectively, compared with that in siNC-transfected cells (Fig 1F). Consistently, the HCV NS3 protein expression level in siRBM24-transfected cells was also significantly decreased (Fig. 1G). Virus propagation in siRBM24-transfected cells was impaired to a similar degree as the decrease in intracellular HCV RNA (Fig. 1H). As a result, the calculated assembly efficiency (the ratio of the number of supernatant HCV RNA copies to that of intracellular HCV RNA copies) and budding efficiency (the ratio of the HCV titer in the cell supernatant to that in cells) were similar in siRBM24- and siNC-transfected cells (Fig. 1I and 1J). These results indicate that the silencing of RBM24 expression reduced HCV replication and that this reduction was not at the assembly or release step of the HCV life cycle.

### RBM24 participates in the translation and replication of HCV

**Figure 2:**
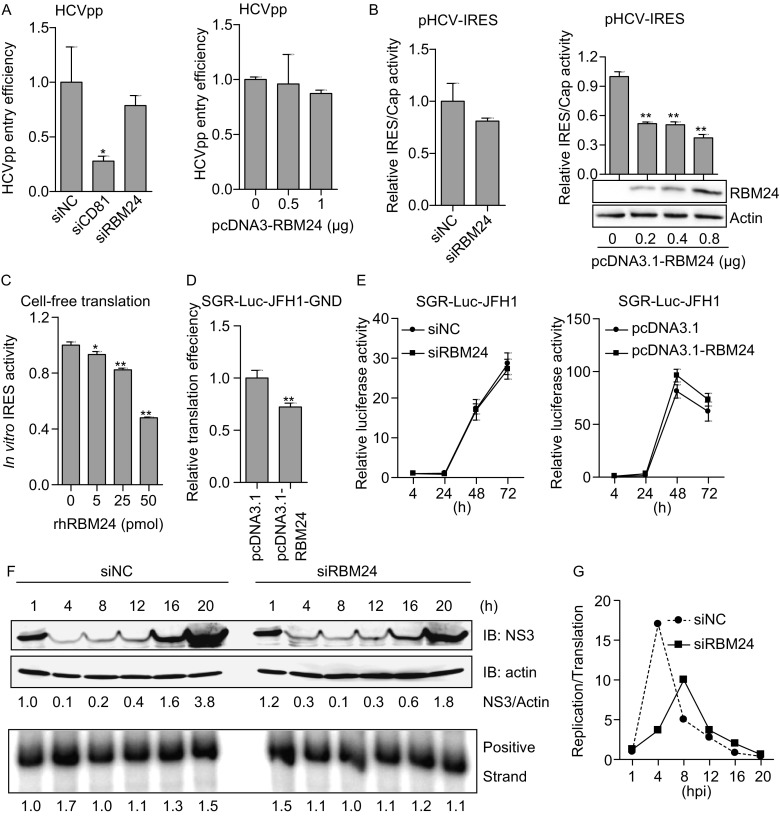
**The involvement of RBM24 in HCV translation and replication**. (A) Huh7.5.1 cells were transfected with the indicated siRNAs or RBM24 expression plasmids and transduced with HCVpp. Cell lysates were collected at 48 h post-transduction, and luciferase activity was measured. (B) Huh7.5.1 cells were transfected with indicated siRNAs or RBM24 expression plasmids together with pHCV-IRES. Cell lysates were collected at 24 h post–transfection, and luciferase assay was performed. HCV IRES-dependent translation relative to cap-dependent translation was calculated and normalized. (C) The RNA fragment containing HCV IRES and the firefly luciferase coding sequence was incubated with either rhRBM24 or BSA in RRL at 30°C for 15 min. Luciferase activity was determined with Steady-Glo®. (D) Huh7.5.1 cells were transfected with pcDNA3.1 or pcDNA3.1-RBM24, and then electroporated with the replication deficiency subgenomic RNA, SGR-Luc-JFH1-GND. Luciferase assay was performed at the indicated time points post-electroporation. (E) Huh7.5.1 cells were transfected with siNC or siRBM24, or a control vector or pcDNA3.1-RBM24 plasmid, and then electroporated with HCV subgenomic RNA, SGR-Luc-JFH1. The replication of HCV RNA was monitored by assessing luciferase expression by luciferase assay at the indicated time points post-electroporation. (F) Huh7.5.1 cells transfected with siNC or siRBM24 were infected with Jc1 at an MOI of 5. The infection was synchronized by incubation at 4°C for 1 h to allow attachment and at 37°C for 1 h to allow virus entry. Total RNA and protein were extracted at the indicated time points post-infection, and the HCV NS3 expression and HCV RNA levels were monitored by Western blotting and RPA, respectively. The NS3 protein level normalized against beta-actin and the HCV RNA level were analyzed by densitometry and are presented as numbers below the figure. (G) The ratio of replication (HCV RNA) to translation (NS3 protein) efficiency was calculated

To determine the step of the HCV life cycle at which RBM24 is involved, the effect of RBM24 on viral entry, translation and subgenomic RNA replication was first studied using HCVpp, an HCV IRES reporter plasmid and a subgenomic replicon system. HCVpp entry efficiency, as indicated by the luciferase activity in HCVpp-transduced Huh7.5.1 cells, was not noticeably altered by RBM24 silencing or overexpression (Fig. 2A), suggesting the lack of influence of RBM24 on HCV entry. pHCV-IRES, a bicistronic reporter plasmid that expresses renilla luciferase in a 5′ cap-dependent manner in addition to firefly luciferase directed by HCV IRES, was used to determine the effect of RBM24 on HCV translation in the context of RBM24 knockdown or overexpression. Though RBM24 silencing didn’t influence the IRES mediated translation (Fig. 2B, left), a dose-dependent inhibition of HCV IRES-dependent translation compared with the cap-dependent translation by RBM24 overexpression was observed (Fig. 2B, right). The inhibition of HCV IRES-dependent translation by RBM24 was further validated by cell-free translation assay with a monocistronic construct composed of HCV IRES and the firefly luciferase coding sequence. In this system, the presence of rhRBM24 protein significantly reduced HCV IRES-mediated translation in a dose dependent manner (Fig. 2C). Thus, RBM24 showed an inhibitory effect on HCV IRES-dependent translation in both the bicistronic and monocistronic constructs. Furthermore, the replication-defective SGR-Luc-JFH1-GND construct with an inactivation mutation (GDD-to-GND) in the active site of NS5B was used to analyze the influence of RBM24 on HCV IRES activity. Huh7.5.1 cells were transfected with pcDNA3.1 or pRBM24 first, and then electroporated with the *in vitro* transcribed RNA (SGR-Luc-JFH1-GND). The intracellular HCV RNA were monitored by realtime RT-PCR immediately after the electroporation and served as input RNA. When normalized by the abundance of input RNA, the luciferase activity at 4 h post electroporation was decreased by RBM24 overexpression, suggesting that RBM24 inhibit the translation efficiency of HCV (Fig. 2D). Taken together, these results indicate that RBM24 is not involved in HCV entry but that it has an inhibitory effect on HCV IRES activity.

Surprisingly, no significant difference in the replication of SGR-Luc-JFH1 was observed between vector- and RBM24-transfected Huh7.5.1 cells or between siNC- and siRBM24- transfected Huh7.5.1 cells (Fig. 2E). Further considering the fact that HCV propagation was reduced in siRBM24-transfected cells (Fig. 1), the inhibition of HCV IRES activity by RBM24 implies that RBM24 is probably required for replication of genomic RNA. Then the profiles of HCV replication and translation were analyzed within 24 hpi in RBM24 knockdown cells. For this purpose, Huh7.5.1 cells transfected with siNC or siRBM24 were infected synchronizationally with Jc1 at an MOI of 5 by sequentially incubation at 4°C for 1 h, washing with PBS and incubation at 37°C for 1 h. HCV NS3 expression and RNA levels were monitored by Western blotting and RPA, respectively, at the indicated time points. In siNC-transfected cells, the NS3 protein expression was detectable at 1 hpi, decreased at 4 hpi, and then gradually increased, and a sharp increase in the NS3 protein level was observed at 16 hpi and 20 hpi, while the HCV RNA level exhibited an increase at 4 hpi, decrease at 8 hpi and then a gradual increase (Fig. 2F). The ratio of replication to translation efficiency peaked at 4 hpi in siNC-transfected cells (Fig. 2G), suggesting that the first round of translation of the released HCV RNA occurred within 1 hpi and that the switch from translation to replication probably took place before 4 hpi. The protein and RNA profiles of HCV were markedly different in siRBM24-transfected cells. First, NS3 protein expression was relatively higher at 1 hpi and 4 hpi in siRBM24-transfected cells compared with that in siNC transfected cells, and its expression was the lowest at 8 hpi; however, its expression was significantly lower at 20 hpi in siRBM24-transfected cells because a sharp increase in NS3 only occurred at 20 hpi. Second, no significant increase in the HCV RNA level was observed at 4 hpi in siRBM24-transfected cells. Third, the peak in the replication to translation ratio was reduced and delayed to 8 hpi. These results suggested that the silencing of RBM24 stimulated the initial translation of HCV proteins but inhibited and delayed replication of HCV genome.

### RBM24 interacts with HCV RNA

**Figure 3:**
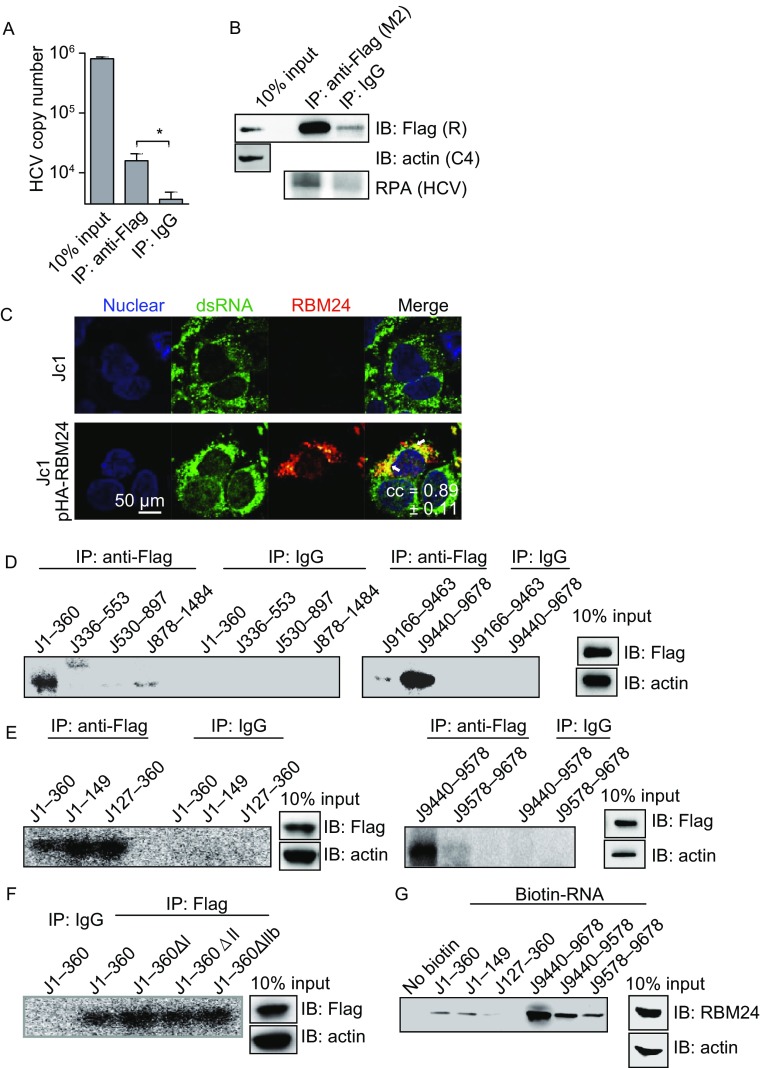
**The interaction between RBM24 and HCV RNA sequences**. (A and B) Huh7.5.1 cells were transfected with pFlag-RBM24 and infected with Jc1 at an MOI of 0.1. The cells were harvested at 72 hpi as described in experimental procedures and immunoprecipitated with either an anti-Flag mouse monoclonal antibody (Flag) or a nonspecific mouse control antibody (IgG). Precipitated HCV RNA was detected by qRT-PCR (A) or RPA (B), and the precipitated NS3, RBM24 and actin were detected by Western blot (B). (C) Huh7.5.1 cells were transfected with vectore or pHA-RBM24 and then infected with Jc1 at an MOI of 1. The localization of double strand RNA and RBM24 protein were detected by immunofluorescence using the J2 antibody and HA antibody respectively. The nuclei were stained with Hoechst 33258. (D–F) A lysate of 293T cells transfected with pFlag-RBM24 was crosslinked with the indicated ^32^P-labeled HCV fragments. The crosslinked nucleotides-proteins were immunoprecipitated with either an anti-Flag mouse monoclonal antibody (Flag) or a nonspecific mouse control antibody (IgG) and detected by autoradiography. The input proteins were detected by Western blot. (G) A lysate of 293T cells transfected with pcDNA3.1-RBM24 was incubated with the indicated biotin-labeled HCV fragments and affinity-precipitated with streptavidin beads. RBM24 protein was detected by Western blot

To determine the manner by which RBM24 is involved in HCV translation and replication, the interaction of RBM24 with HCV RNA was first examined. RIP was performed using a lysate of Jc1-infected Huh7.5.1 cells in which Flag-RBM24 was over-expressed. The precipitant was analyzed by qRT-PCR or RPA. As shown in Fig. 3A and 3B, a significant amount of HCV RNA was co-immunoprecipitated with Flag-RBM24 by a Flag tag antibody compared with IgG control, suggesting the association of RBM24 with HCV genomic RNA in HCVcc-infected cells. Further, RBM24 co-localized with HCV double strand RNA (dsRNA) in cytoplasm in Jc1 infected Huh7.5.1 cells (Fig. 3C) as shown by immunofluorescence detection, supporting the association of RBM24 with HCV RNA.

Non-specific CLIP was then performed to identify the RBM24 binding sequences in HCV RNA. Various HCV RNA fragments labeled with ^32^P were subjected to CLIP together with the Flag-RBM24 containing cell lysate. Fragments of the HCV 5′-UTR (J1–360) and 3′-UTR (J9440–9678), but not of the regions containing Core (J336–553, J530–897, and J878–1484) or the NS5B coding region elements (J9116–9463), were strongly crosslinked to Flag-RBM24 (Fig. 3D). In further CLIP analysis, the 5′-UTR region was divided into 2 fragments, J1–149 (SL I-SL II) and J127–360 (SL III-SL IV), according to the secondary structure, and the 3′-UTR region was divided into J9440–9578 (VSL2-polyU tract) and J9578–9678 (3′-X region). The results shown in Fig. 3E indicate that nt 1–149, nt 127–360 and nt 9440–9578 in the HCV RNA 5′-UTR and 3′-UTR regions interacted with Flag-RBM24. Neither deletion of nt 5–19 corresponding to SLI (J1–360ΔI), nt 43–119 corresponding to SLII (J1–360ΔII) nor nt 65–100 corresponding to the upper part of SLII (J1–360ΔIIb) could abolish the crosslinking of the HCV 5′-UTR to Flag-RBM24 (Fig. 3F).

The interactions of RBM24 with the HCV 5′-UTR and 3′-UTRs were further validated by biotin pull-down assay. Different fragments of the 5′-UTR and 3′-UTRs were labelled with biotin and incubated with a lysate of 293T cell overexpressing RBM24. Biotinylated RNA and bound proteins were then isolated on streptavidin agarose and analyzed by Western blotting with an RBM24 antibody. Consistent with the results of CLIP assay, the fragments J1–360, J1–149, J9440–9678 and J9440–9578 efficiently pulled down the RBM24 protein (Fig. 3G). Interestingly, RBM24 was also coprecipitated with the 3′-X fragment, which did not crosslink to Flag-RBM24 as shown by CLIP assay (Fig. 3F). Taken together, these results demonstrated that RBM24 interacted with the HCV 5′-UTR and 3′-UTR (nt 9440–9578).

### RBM24 blocks 80S ribosome assembly on HCV IRES

**Figure 4:**
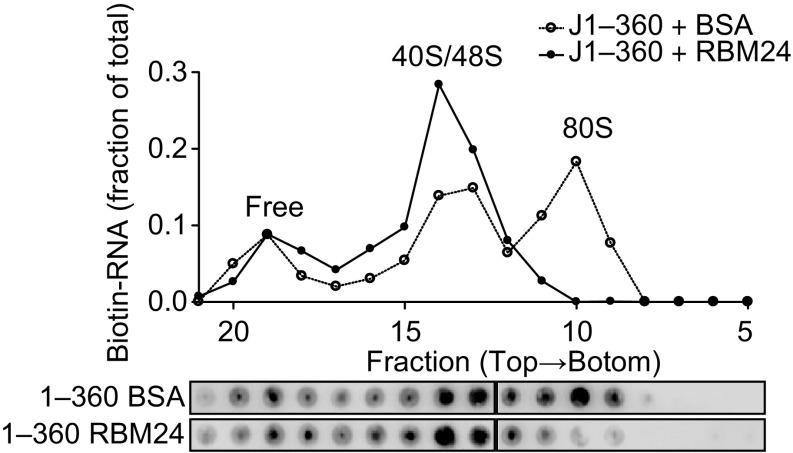
**RBM24 blocks 80S ribosome assembly on HCV IRES**. Biotin-labeled JFH1 nt 1–360 together with rhRBM24 or BSA were incubated in RRL at 30°C for 15 min. The ribosome complexes were separated by sucrose gradient ultracentrifugation. The distribution of biotin-RNA was detected by dot-blot assay with streptavidin-HRP and analyzed by densitometry (fractions 5–21 were shown)

HCV IRES-mediated translation is initiated by 80S ribosomes, which are sequentially formed by 40S subunit binding, eIF3 and ternary complex binding, GTP hydrolysis, eIF release, and 60S subunit binding (Lukavsky, 2009). To elucidate the mechanism underlying the inhibition of HCV IRES activity by RBM24, ribosome assembly was examined in a rabbit reticulocyte lysate (RRL) system. Biotin-labelled HCV IRES (J1–360) RNA together with rhRBM24 protein or BSA control were incubated with RRL, and the resulting ribosomal complexes were separated by sucrose density gradient ultracentrifugation and analyzed by dot blot. Consistent with previous reports, 80S ribosome formation was observed after 15 min of incubation in the BSA control group, whereas in the presence of recombinant RBM24, the 80S peak was significantly lower, and most of the RNA was retained in the 40/48S peaks (Fig. 4). Thus, the inhibitory effect of RBM24 on HCV IRES activity was due to the inhibition of 80S ribosome assembly occurring at the step after 40S ribosome binding.

### RBM24 affects the interaction of Core protein with HCV IRES

**Figure 5:**
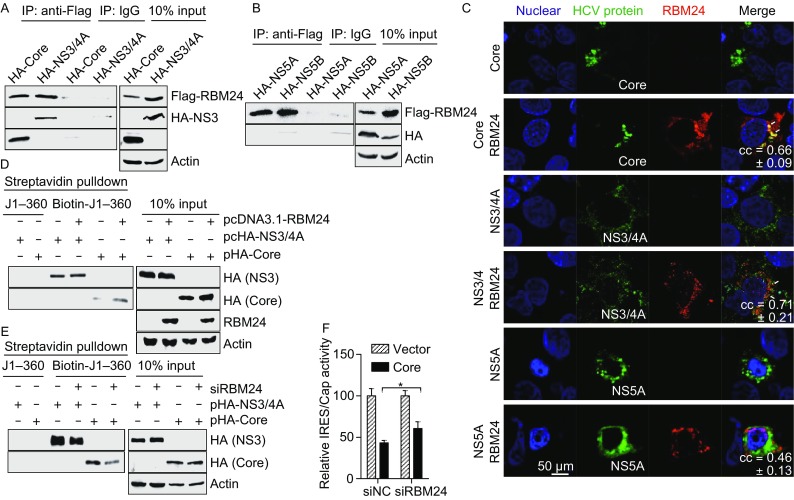
**The interactions between RBM24 and HCV proteins**. (A) 293T cells were transfected with Flag-RBM24 together with HA-Core or HA-NS3/4A. Cell lysates were immunoprecipitated with either an anti-HA mouse monoclonal antibody (HA) or a nonspecific mouse control antibody (IgG) in the presence of RNase A. Immunoprecipitated proteins were detected with an anti-HA rabbit monoclonal antibody (HA) or an anti-DYKDDDDK rabbit polyclonal antibody (Flag) correspondingly. (B) Co-IP was performed with the lysate of 293T cells transfected with pFlag-RBM24 and pHA-NS5A, or with pFlag-RBM24 and pHA-NS5B. (C) Huh7.5.1 cells were transfected with HCV protein (Core, NS3/4A or NS5A) expression plasmids together with pHA-RBM24. The localization of Core, NS3/4A, NS5A and RBM24 was detected by immunostaining with HA and Flag antibodies. The nuclei were stained with Hoechst 33258. (D) 293T cells were co-transfected with pcDNA3.1-RBM24 with either pHA-Core or pHA-NS3/4A. The cell lysates were incubated with the indicated biotin-labeled HCV fragments and affinity-precipitated with streptavidin beads. Precipitated proteins were detected by Western blot with the corresponding antibodies. (E) Huh7.5.1 cells were transfected with siRBM24 as described in experimental procedures and then transfected with either pHA-Core or pHA-NS3/4A. Cell lysates were incubated with the indicated biotin-labeled HCV fragments and affinity precipitated with streptavidin beads. Precipitated proteins were detected by WB with the corresponding antibodies. (F) Huh7.5.1 cells were transfected with indicated siRNAs first then transfected with pHCV-IRES and pHA-Core. Luciferase assay was performed 24 h later

Viral and host factors interact and work together to control HCV replication. Moreover, the HCV structural protein Core and non-structural protein NS3 have been reported to bind to the HCV IRES as RBM24. Thus we next analyzed the interplay between RBM24, HCV proteins and the HCV IRES. Interestingly, RBM24 interacted with the HCV Core and NS3 proteins but not the NS5A or NS5B proteins, as shown by co-immunoprecipitation assay (Fig. 5A and 5B). Consistently, immunofluorescence results showed that RBM24 was partially co-localized with HCV Core and NS3 protein, while no obvious colocalization of RBM24 with NS5A was found in Huh7.5.1 cells (Fig. 5C). Both the Core and NS3 proteins have been proven to be trans-modulating factors in HCV replication (Shimoike et al., 1999; Ray and Das, 2011). Thus we then analyzed whether RBM24 influences the interactions of the HCV Core and NS3 proteins with the HCV IRES. To this end, we performed biotin pull-down assay with a biotinylated HCV 5′-UTR (J1–360) and a cell lysate containing Core or NS3 in the presence or absence of RBM24. As shown in Fig. 5D, the co-expression of RBM24 with the Core and NS3 proteins did not influence their expression. However, the amount of HA-Core precipitated with the HCV 5′-UTR was significantly increased by the overexpression of RBM24, while the amount of HA-NS3 coprecipitated with the HCV 5′-UTR was not affected. In addition, the silencing of RBM24 expression in Huh7.5.1 cells significantly decreased the amount of HA-Core and HA-NS3 coprecipitated with the HCV 5′-UTR (Fig. 5E). The effect of Core on HCV IRES activity was detected in the background of siRBM24 transfection. Results showed that the knockdown of RBM24 expression slightly reduced the inhibitory effect of Core on IRES activity (Fig. 5F). These results suggested that RBM24 regulates the interaction between the Core and the HCV 5′-UTR.

### RBM24 enhances the interaction between the HCV 5′- and 3′-UTRs

**Figure 6:**
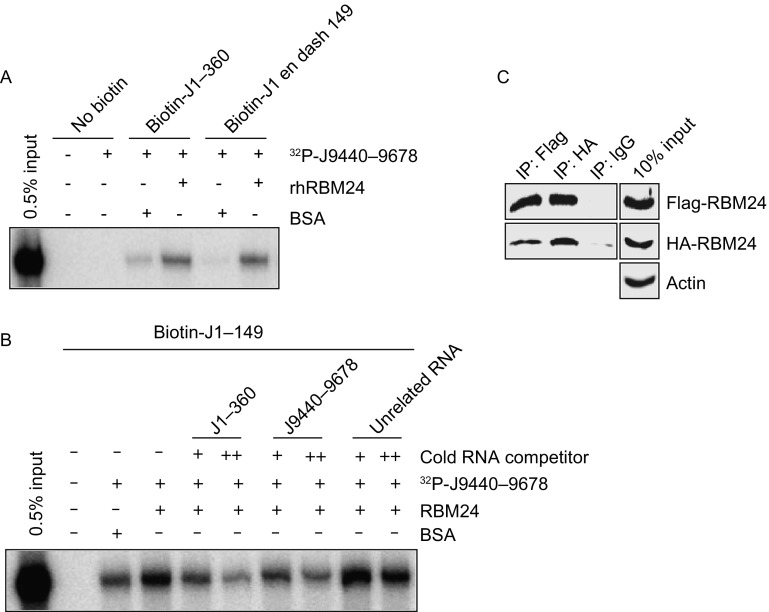
**RBM24 enhances the interaction between the HCV 5′- and 3′-UTRs**. (A) Interaction between the 5′-UTR (J1–360 or J1–149) and 3′-UTR (J9440–9678) was analyzed in the presence or absence of rhRBM24, as described in the experimental procedures. Precipitated RNAs were analyzed on a 6% urea-PAGE gel and by autoradiography. (B) The effects of cold RNA competitors (J1–360 and J9440–9678) on the interaction between HCV J1–149 and J 9440–9678 were analyzed. (C) The 293T cells were co-transfected with pFlag-RBM24 and pHA-RBM24. Cell lysates were immunoprecipitated with either an anti-Flag mouse monoclonal antibody (Flag), an anti-HA mouse monoclonal antibody (HA) or a nonspecific mouse control antibody (IgG). Precipitated proteins were detected with an anti-HA rabbit monoclonal antibody (HA) or an anti-DYKDDDDK rabbit polyclonal antibody (Flag) correspondingly

In addition to the 5′-UTR, RBM24 can bind to the 3′-UTR of HCV RNA (Fig. 3). The long-range RNA-RNA interaction of the 5′- and 3′-UTRs which is mediated by direct interaction of the 5′- and 3′-UTRs or by *trans* factors, is thought to play a role in viral translation modulation and in the switch from protein synthesis to RNA replication. Thus it is possible that RBM24 participates in translation and replication by enhancing the interaction of the HCV 5′-UTR and 3′-UTRs. To test this hypothesis, this interaction was assessed by streptavidin pull-down assay. Streptavidin beads were coated with a biotinylated HCV 5′-UTR and then incubated with a ^32^P-labeled 3′-UTR in the presence or absence of recombinant RBM24. The amount of 3′-UTR RNA that interacted with the coated beads was analyzed by urea-PAGE. The results showed that the interactions of J1–360 and J1–149 with J9440–9678 were significantly enhanced by rhRBM24 compared with the unrelated protein BSA (Fig. 6A). Competition experiments revealed that the interaction between biotin-J1–149 and ^32^P-J9440–9678 enhanced by RBM24 could be out-competed by increasing the amount of cold J1–149 or J9440–9678 but not that of unrelated RNA (Fig. 6B), indicating the specificities of the interactions between RBM24 and these HCV RNA fragments.

RBM24 only has one RRM domain, and the manner by which it interacts with both the 5′- and 3′-UTRs and enhances their interaction is unknown. RBM24 has an alanine-rich domain containing 2 uninterrupted polyalanine stretches at the C-terminus which is thought to mediate oligomerization and self-aggregation (Measey et al., 2009). Indeed, Flag- and HA-RBM24 co-immunoprecipitated when they were co-expressed in 293T cells (Fig. 6C), suggesting a potential self-interaction of RBM24. Hence, RBM24 is a *trans*-acting factor that enhances the interaction between the 5′-UTR and 3′-UTRs of the HCV genome; therefore it may participate in the control of HCV replication.

## Discussion

After being released into the cytoplasm, the genomic RNA of HCV is first used as template for protein synthesis. Once viral proteins are synthesized, the viral RNA is replicated, beginning with negative-strand RNA synthesis using the genomic RNA as a template. The fine-tuned regulation of the switch from translation to replication has still not been fully elucidated. In this study, we identified RBM24 as a host factor involved in both translation and RNA replication in HCV life cycle. First, RBM24 inhibits the HCV IRES activity by binding to the 5′-UTRs of HCV RNA and impairing 80S ribosome assembly on the HCV IRES. RBM24 also enhances the interaction of Core and 5′-UTRs of HCV RNA, which suppress the expression of HCV. Furthermore, RBM24 enhances the interaction between the HCV 5′- and 3′-UTR that is required for efficient RNA replication and therefore may function at the switch from translation to replication.

HCV protein translation is mediated by the highly conserved IRES structure and 80S ribosome assembly on the IRES is a process including multiple steps that are mediated by specific IRES structural domains. The SLII domain is of great interest because it is indispensable for the conformational change of the 40S subunit induced by IRES binding (Spahn et al., 2001; Filbin and Kieft, 2011; Boehringer et al., 2005). Interaction of the SLII domain with ribosomal protein 5 (RPS5) is required for 80S ribosome assembly (Bhat et al., 2015), and has been reported to promote eIF2-GDP release. Further, deletion of this domain blocks 80S assembly after 48S complex formation (Locker et al., 2007). RBM24 blocks the formation of the 80S ribosome complex after 40/48S complex formation, similar to what occurs following SLII domain deletion. It is likely that the binding of RBM24 to the 5′-UTR does not influence the interaction of the IRES with 40S ribosome but it does interfere with the 80S ribosome assembly.

Besides affecting the 80S ribosome assembly, RBM24 may influence HCV IRES activity through interaction with HCV proteins. HCV Core not only functions as a structural nucleocapsid protein but also inhibits the expression of HCV (Shimoike et al., 1999; Shimoike et al., 2006; Tanaka et al., 2000). Here we found that RBM24 interacts with both 5′-UTR of HCV RNA and Core protein, and enhances the interaction of Core and 5′-UTR of HCV, which is thought to be a mechanism of translation inhibition (Shimoike et al., 1999). It has been reported that HCV NS3 binds to the SLIV and hinders the interaction of La protein with HCV IRES, resulting in inhibition of HCV-IRES activity (Ray and Das, 2011). Although we found that RBM24 interacted with NS3 protein, its presence did not influence the interaction of NS3 and the 5′-UTR. Thus, RBM24 may also suppress the expression of HCV through its interaction with Core but not NS3 protein.

Long-range RNA-RNA interactions of HCV RNA either mediated directly or by protein-RNA interactions are required for efficient replication and play a crucial role in switching among different steps of the HCV life cycle, such as the translation-replication and replication-assembly switches (Shetty et al., 2013). RBM24 binds to both the 5′-UTR and 3′-UTRs of HCV, and although it contains only one RNA-binding domain, it has the ability to form dimers or oligomers. As a result, it could act as a protein bridge to facilitate long-range RNA interactions between the 5′- and 3′-UTRs of HCV genome. Knockdown of the endogenous expression of RBM24 in Huh7.5.1 cells enhances and prolongs translation and delays the translation-replication switch during the early events after virus entry (Fig. 2F and 2G); thus RBM24 is thought to inhibit translation and promote replication and to play a role in this switch. As mentioned above, the translation and replication of HCV are regulated in complex manners by multiple factors, including *cis*- and *trans*-factors, the most studied of which is La protein. According to previous reports, multi-round translation of HCV RNA is enhanced by La protein (Kumar et al., 2013) as the HCV RNA genome is released into the cytoplasm. When the viral protein accumulates to a threshold in the presence of NS3 protein, La-mediated enhancement of translation is inhibited, and La protein promotes the replication of HCV RNA by facilitating interaction of the 5′- and 3′-UTRs (Ray and Das, 2011). Furthermore, RNA-binding proteins have been demonstrated to be interacted with each other, for example, La protein and PCBP2 form an oligomer, and PCBP2 and polypyrimidine tract binding protein (PTB) interact with La protein (Fontanes et al., 2009). Very recently, another RNA binding protein, HuR was shown to interact with 3′-UTR of HCV genome, compete with PTB but facilitate La binding to the 3′-UTR (Shwetha et al., 2015). In contrast with La protein, RBM24 is thought to inhibit translation but to facilitate the HCV 5′- and 3′-UTR interaction required for multi-round replication. The fact RBM24 silencing only reduced HCV replication in HCVcc system but not in subgenomic replicon cells implied that the modulation of HCV replication by RBM24 requires HCV structure proteins especially the Core protein. It will be interesting to reveal whether RBM24 interacts with La protein as well as other *trans*-acting factors, in addition to how RBM24 and these proteins function together during the replication-translation switch.

Although the expression of RBM24 has been shown to be relatively low in the liver in previous reports (Miyamoto et al., 2009), its mRNA level in HCV infected Huh7.5.1 cells could rise to a high level in this study compared with the copy number of the house keeping gene actin. Meanwhile, we observed high signal intensity in the liver with inflammation by immunohistochemistry (data not shown). Thus, the up-regulation of RBM24 by HCV could be a consequent of HCV induced inflammatory response and the balance of HCV translation and replication controlled by RBM24 might be involved in the establishment or maintenance of viral persistence, which is a major characteristic of HCV infection.

Previous reports have shown that RBM24 regulates the stability and/or alternative splicing of the mRNAs of different genes. However, overexpression of RBM24 did not affect the stability of HCV RNA (data not shown). Thus, RBM24 regulate the viral protein expression in a novel way. The binding of RBM24 to RNA could be either sequence-dependent or structure-dependent, and it could interact with different RNAs and possibly be involved in the replication of other viruses, as have been reported for the binding of PTB and La protein to the RNAs of encephalomyocarditis virus (EMCV), Japanese encephalitis virus (JEV) and HBV (Vashist et al., 2011; Vashist et al., 2009; Heise et al., 1999; Horke et al., 2002; Kim and Jeong, 2006; Kaminski and Jackson, 1998; Zang et al., 2001). Indeed, our results demonstrate the involvement of RBM24 in the regulation of VSV and HBV replication (data not shown). Thus, it could be a common regulator for virus replication.

In summary, we identified RBM24 as a host factor involved in both translation and RNA replication in HCV life cycle. RBM24 binds to both the 5′- and 3′-UTRs of HCV RNA, impairing 80S ribosome assembly on the HCV IRES and enhancing the interaction between the HCV 5′- and 3′-UTR that is required for efficient RNA replication and therefore may function at the switch from translation to replication.

## Materials and methods

### Cell culture

Huh7.5.1 cells (kindly provided by Prof. Frank Chisari) and 293T cells were cultured in Dulbecco’s modified Eagle medium (DMEM) with 2 mmol/L of glutamine (Gibco®, 12100-046),10% fetal bovine serum (FBS) (Gibco®, 10099-141) and 100 U/mL of penicillin-streptomycin (Gibco®, 15140-122) at 37°C in a 5% CO_2_ atmosphere.

### Virus production and titration

The chimeric construct, Jc1 (kindly provided by Prof. T. Pietschmann and Prof. R. Bartenschlager), was linearized, *in vitro*-transcribed and electroporated into Huh7.5.1 cells as previously described (Pietschmann et al., 2006). The virus in the supernatant was concentrated and purified by PEG 8,000 and ultracentrifugation sequentially, and then titered by immunofluorescence staining with NS3 antibody 8 G-2 (Abcam®, ab65407) (Jones et al., 2011). The J399EM virus which is derived from the JFH-1 virus (kindly provided by Takaji Wakita) by inserting EGFP into the NS5A, was prepared as previously described (Zhu et al., 2014). The HCV pseudoparticle (HCVpp) was generated by transfection of 293T cells with pNL4.3.lucR-E- and pcDNA3.1-E1E2 (a gift from Jin Zhong) plasmids (Zhu et al., 2014).

To measure the intracellular virus titers, cells were washed and collected in 200 μL PBS and lysed by 3 rounds of freezing and thawing. Jc1 virus was titrated by immunostaining with the NS3 antibody as described by Jones et al. (2011). Briefly, Huh7.5.1 cells were seeded in 8-well chamber slides and infected with 10-fold-serially diluted virus. The infectious medium was removed and replaced with fresh medium 4 h after infection. 72 h post infection, cells were fixed and stained with anti-NS3 antibody. Viral titers were expressed as the number of focus-forming unit (FFU) per mL. J399EM was titrated by endpoint dilution assay using EGFP as indicator (Zhu et al., 2014).

### Plasmid construction

The HCV protein expression plasmids pXJ40-HA-Core, pXJ40-HA-NS3/4A, pXJ40-HA-NS5A and pXJ40-HA-NS5B have been described previously (Guo et al., 2014). The ORF of human RBM24 transcript 001 (ENST00000379052) was amplified using cDNA from HeLa cells and cloned into pcDNA3.1(−), pXJ40-Flag or pXJ40-HA respectively, to generate eukaryotic expression plasmids,pcDNA3.1-RBM24, pXJ40-Flag-RBM24 and pXJ40-HA-RBM24. A prokaryotic RBM24 expression plasmid pET33b-EK-RBM24 was generated by cloning the coding sequence of RBM24 into pET33b (+). All of the constructs were validated by sequencing.

### Prokaryotic expression and purification of recombinant human RBM24

*Escherichia coli* BL21(DE3) cells were transformed with pET33b-EK-RBM24. A positive monocolony was inoculated into 1 L of LB medium containing 50 μg/mL of kanamycin and grown at 37°C in a shaker until the A600 reached 0.6. Cells were induced with 0.5 mmol/L isopropyl β-D-1-thiogalactopyranoside for 4 h at 37°C and were then harvested by centrifugation and resuspended in low imidazole buffer (300 mmol/L NaCl, 50 mmol/L Tris (pH 8.0), 0.02% NaN_3_, 20% glycerol, and 10 mmol/L imidazole) with 0.5% IGEPAL® and 0.07% β-ME. Next, the cells were lysed using an ultrasonic homogenizer, and the lysate was clarified by centrifugation and filtration through a 0.22 μmol/L syringe filter. The supernatant was loaded onto a column containing 10 mL Ni-NTA His∙Bind® resins (Novagen, 70666). After washing sequentially with 10 mmol/L, 50 mmol/L and 100 mmol/L imidazole buffer (20, 10, and 10 column volumes, respectively), the protein was eluted with 1.5 column volumes of 500 mmol/L imidazole buffer and circulated at 37°C for 15 min. After the protein was dialyzed into enterokinase buffer (20 mmol/L Tris (pH 8.0), 50 mmol/L NaCl, 2 mmol/L CaCl_2_, and 10% glycerol), the tag was removed by enterokinase (New England Biolabs, P8070L) digestion followed by Trypsin inhibitor-Agarose (Sigma-Aldrich®, T-0637) and His∙Bind resin incubation to remove the enterokinase and potentially undigested protein. Tag-free recombinant human RBM24 (rhRBM24) was then dialyzed in RNA binding buffer, concentrated with PEG 20,000, passed through a 0.22 μm syringe filter, aliquoted and stored in liquid nitrogen.

### Plasmid transfection and RNA interference

Plasmids and siRNAs were transfected into cells using Lipofectamine 2000 (Invitrogen™, 11668-019), according to the manufacturer’s instructions. The following siRNAs were used: AllStars negative control siRNA (siNC, QIAGEN, SI03650318), siHCV (target sequence: 5′-GGUCUCGUAGACCGUGCAC-3′) and siRBM24 (Qiagen, SI03030195). Because transfection of siRBM24 once reduces the RBM24 mRNA level but not the protein level, transfection was performed twice as previously described (Zhu et al., 2014).

### RNA extraction and quantitative real-time RT-PCR (qRT-PCR)

RNA was extracted with TRIzol, and specific RNAs of interest were quantified with a QuantiTect SYBR Green RT-PCR Kit (Qiagen, 204243), following the manufacturer’s instructions. The primers for HCV and actin have been described previously (Zhu et al., 2014), and those for RBM24 included RBM24-SybrG-F and RBM24-SybrG-R are listed in Table 1.

**Table 1:** **Primers used in the study**

	Oligonucleotide	Sequence
Primers for RBM24	RBM24-SybrG-F	5′-GGCCAACGTGAACCTGGCATACTT-3′
	RBM24-SybrG-R	5′-GGCAGGTATCCCGAAAGGTCTTTGT-3′
Primers for HCV RNA fragments	EcT7-Δ5′I-F	5′-TGAGGAATTC*TAATACGACTCACTATA*GACCTGACACTCCGCCATGAATC-3′
	J1390-R	5′-CCCGCTAACGATGTCTATGATGACCTCG-3′
	Δ5′II-F	5′-CTCCGCCATGAATCACTCCCCCCCCCCTCCCGGGAG-3′
	Δ5′II-R	5′-CTCCCGGGAGGGGGGGGGGAGTGATTCATGGCGGAG-3′
	EcT7G/FH5-F	5′-TGAGGAATTC*TAATACGACTCACTATA*GACCTGCCCCTAATAGGGGCGA-3′
	Δ5′IIb-F	5′-GTGAGGAACTACTGTCTTCGTCGTACAGCCTCCAGGCCCCCCCC-3′
	Δ5′IIb-R	5′-GGGGGGGGCCTGGAGGCTGTACGACGAAGACAGTAGTTCCTCAC-3′
	J127-T7-F	5′-*TAATACGACTCACTATAGGGACT*CCCGGGAGAGCCATAGTGGTCTG-3′
	J149-R	5′-CAGACCACTATGGCTCTCCCGGG-3′
	J336-T7-F	5′-*TAATACGACTCACTATAGGGACT*GCACCATGAGCACAAATCCTAAACC-3′
	J360-R	5′-GGTTTAGGATTTGTGCTCATGGTGC-3′
	J530-T7-F	5′-*TAATACGACTCACTATAGGGACT*CCCATCCCCAAAGATCGGCGCTCC-3′
	J553-R	5′-GGAGCGCCGATCTTTGGGGATGGG-3′
	J875-T7-F	5′-*TAATACGACTCACTATAGGGACT*GGCCCTGTTGTCCTGCATCAC-3′
	J897-R	5′-GTGATGCAGGACAACAGGGCCAG-3′
	J1484-R	5′-CCACCCCAGCGGCCAGCAGAAGG-3′
	J9166-T7-F	5′-*TAATACGACTCACTATAGGGACT*GAAGAGTCGGGCTCGCGCAGTCAGG-3′
	J9440-T7-F	5′-*TAATACGACTCACTATAGGGACT*AGAGCGGCACACACTAGGTACACTCC-3′
	J9463-R	5′-GTGTACCTAGTGTGTGCCGCTC-3′
	J9578-T7-F	5′-*TAATACGACTCACTATAGGGACT*TGGTGGCTCCATCTTAGCCCTAG-3′
	J9578-R	5′-GAAAGAAAGTAGAATAAGATGAGAAGGG-3′
	J9678-R	5′-ACATGATCTGCAGAGAGACCAGTTACGGC-3′
Primers for RPA probes	J5025-T7-F	5′-T*AATACGACTCACTATAGGGACT*GGGAGGCAGTTTTCACCGGCC-3′
	J5025-F	5′-GGGAGGCAGTTTTCACCGGCC-3′
	J5350-T7-R	5′-T*AATACGACTCACTATAGGGACT*GACTCCTCCAGCTAGGACCCACGTGC-3′
	J5350-R	5′-GACTCCTCCAGCTAGGACCCACGTGC-3′

The T7 promoter sequence was marked as italic, and the restriction enzyme sites are shown by underline

### Western blot, co-immunoprecipitation (Co-IP) and immunofluorescence analyses

The procedure for Western blotting, co-immunoprecipitation and immunofluorescence was the same as previously described (Xu et al., 2012), noting that 200 μg of total protein was used to detect endogenous RBM24. The following antibodies were used: rabbit anti-RBM24 (Abcam®, AB94567), rabbit anti-HA (Cell Signaling Technology®, #3724) and DYKDDDDK (Cell Signaling Technology®, #2368), mouse anti-HA (Sigma-Aldrich®, H9658) and mouse anti-FLAG® (Sigma-Aldrich®, F1804), and mouse monoclonal antibody (MAb) J2 (Scion, 10010500). For RNase A treatment in the Co-IP procedure, 20 μg/mL of RNase A was added to the cell lysate for IP and incubated overnight at 4°C.

### *In vitro* transcription, *in vitro* translation and ribosome assembly assay

First, the DNA templates for *in vitro* transcription were produced by PCR amplification of pJFH1 with the corresponding T7-tagged forward primers and the reverse primers listed in Table 1. HCV RNA fragments were then generated by *in vitro* transcription of these DNA templates with a MEGAscript® T7 Transcription Kit (Invitrogen™, AM1334). Biotin-11-UTP (Invitrogen™, AM8450) or [α-^32^P]-UTP (Perkin Elmer, NEG507T250UC) was added to the reaction as required to produce labeled RNA. These RNA fragments were then denatured, digested with TURBO™ DNase (Ambion®, AM2238) at 37°C for 30 min, purified with TRIzol and dissolved in appropriate buffer.

The template for *in vitro* translation was generated according to the sequence of pSGR-Luc-JFH1 (Cao et al., 2014) between the *Eco*RI and *Pme*I sites. A rabbit reticulocyte lysate (RRL, Promega, L4960)-based translation reaction system was established as previously described (Bai et al., 2013). Briefly, a total of 0.5 μg of template RNA and 5 to 50 pmol of rhRBM24 or a non-specific control protein, BSA, were added to 150 μL of the reaction system and incubated at 30°C for 15 min. The reaction was halted with 1× Passive Lysis Buffer and luciferase activity was immediately determined with Steady-Glo (Promega, E2520).

For ribosome assembly assay, 1 μg of biotin-labeled HCV IRES RNA (J1–360) and 12.5 pmol of rhRBM24 or BSA were added to a ribosome assembly mixture based on RRL. The mixtures were incubated at 30°C for 15 min. The reaction was halted, and the mixtures were analyzed with 10%–40% sucrose gradients by ultracentrifugation, as previously described (Filbin et al., 2013). The gradients were fractionated into 22 fractions, and 200 μL of each fraction was blotted onto an Amersham Hybond™-N+ membrane with a Whatman® Minifold® I 96 well dot-blot array system. The membrane was crosslinked with a HL-2000 Hybrilinker at 1,200 J, and the blotted biotin-RNA was detected with Streptavidin-HRP (U-Cytech, CT353) and SuperSignal® West Pico Chemiluminescent Substrate.

### RNA immunoprecipitation (RIP)

RIP was performed as previously described with minor optimizations (Keene et al., 2006). Briefly, Huh7.5.1 cells transfected with pXJ40-Flag-RBM24 and infected with Jc1 were lysed in polysome lysis buffer (100 mmol/L KCl, 5 mmol/L MgCl_2_, 10 mmol/L HEPES (pH 7.0), 0.5% IGEPAL®, 1 mmol/L DTT, 100 units/mL RNasin®, 400 μmol/L Ribonucleoside Vanadyl Complex (New England Biolabs®, S1402S) and complete protease inhibitor cocktail (Roche)) and inverted for 30 min at 4°C, and the cell lysate were then centrifuged at 12,000 ×*g* for 15 min to remove cell debris. The cell lysate containing 800 μg total protein was incubated with protein G beads pre-coated with mouse anti-FLAG (Sigma-Aldrich, F1804) or a non-specific control antibody at 30°C for 4 h. After washing 5 times with NT-2 buffer (50 mmol/L Tris-HCl (pH 7.5), 150 mmol/L NaCl, 1 mmol/L MgCl_2_, and 0.05% IGEPAL®), precipitated RNA was extracted with TRIzol for subsequent analysis.

### UV crosslinking immunoprecipitation (CLIP)

Total protein was prepared from 293T cells transfected with Flag-RBM24 using polysome lysis buffer without RNasin. ^32^P-labeled RNA fragments (2000 cps) were incubated with 100 μg total protein for 20 min and then UV crosslinked with a HL-2000 Hybrilinker on ice for 10 min. 400 μL IP buffer containing 30 μg RNase A and 30 U RNase ONE™ were added to each reaction and incubated at 37°C for 1 h. The mixtures were incubated with protein G beads coated with the indicated antibody at 30°C for 4 h. The beads were then washed 5 times with IP buffer, incubated at 95°C for 10 min with 2× Laemmli buffer and subjected to SDS-PAGE. The gel was dried with a Model 583 gel dryer (Bio-Rad) and autoradiographed on a phosphor screen (Perkin Elmer, 7001722) for 1–7 days. Signal was collected with a Cyclone® Plus and analyzed with OptiQuant™.

### RNase protection assay (RPA)

^32^P-labeled probes for HCV positive-strand and negative-strand detection were prepared by *in vitro* transcription and dissolved in 1× RPA Buffer (40 mmol/L PIPES (pH 6.4), 1 mmol/L EDTA (pH 8.0), 400 mmol/L NaCl, and 80% formamide). Standard RPA was performed to detect positive-strand HCV RNA (Guan et al., 2011). Total RNA was incubated with ^32^P-labeled probes and hybridized in hybridization buffer by incubation overnight at 51°C after denaturation at 95°C for 15 min. RNase digestion was performed with 300 μL of RNA digestion mix (500 mmol/L NaCl, 10 mmol/L Tris (pH 7.5), 5 mmol/L EDTA, 350 U/mL RNase T1, and 4.5 μg/mL RNase A) at 30°C for 1 h. Reactions were stopped by the addition of 50 μg of proteinase K and 20 μL 10% SDS. The samples were precipitated with ethanol and glycogen (Thermo Scientific, R0551) and analyzed by 6% urea-PAGE. The gel was dried and autoradiographed as described above.

### Streptavidin pulldown

The biotin-RNA fragments of interest dissolved in RNA folding buffer (100 mmol/L KCl, 20 mmol/L HEPES (pH 7.6), and 5 mmol/L MgCl_2_) were folded by incubation at 70°C for 2 min and cooled at RT. The total protein (400 μg) of interest was incubated with 50 pmol of the indicated folded biotin-RNA fragments, 40 U of RNasin and 50 μg of yeast tRNA (Ambion, AM7119) at 30°C for 30 min. Dynabeads M-280 Streptavidin (Invitrogen™, 11205D) were resuspended with 400 μL of RNA binding buffer after being sequentially washed with 1 mL each of solution A (0.1 mol/L NaOH and 0.05 mol/L NaCl), solution B (0.1 mol/L NaCl) and RNA binding buffer (100 mmol/L KCl, 20 mmol/L HEPES (pH 7.6), 5 mmol/L MgCl_2_, 10% glycerol, 1 mmol/L DTT, 0.1% IGEPAL®, and 400 μmol/L RVC), with two washes in each solution. The aforementioned motioned protein-RNA mixtures were then added to the beads and incubated at 30°C for 2 h. The beads were washed 5 times with RNA binding buffer by magnetic separation, incubated at 95°C for 10 min with 2× Laemmli buffer, and subjected to Western blotting.

### 5′-, 3′-UTR co-precipitation assay

5′-, 3′-UTR co-precipitation assay was performed as previously described with minor optimizations (Wang et al., 2011). Briefly, biotin-RNA was incubated with 25 μL streptavidin beads at 30°C for 30 min. ^32^P-labeled RNA probes were incubated with BSA or rhRBM24 in the presence of 10 μg tRNA (and cold competitor RNA for the experiments described in Fig. 5C) at 30°C for 30 min in RNA binding buffer. The mixtures were then incubated with RNA-coated streptavidin beads at 30°C for 2 h. The beads were collected, washed, and incubated in Gel Loading Buffer II (Ambion®, AM8546G) at 95°C for 10 min. The precipitated RNA was then analyzed with a 6% urea-PAGE gel and autoradiographed

### Statistical analysis

The data were analyzed using the two-tailed unpaired *t*-test. Statistical significance was set at a *(*P* < 0.05) or **(*P* < 0.01).
